# Selecting dissimilar genes for multi-class classification, an application in cancer subtyping

**DOI:** 10.1186/1471-2105-8-206

**Published:** 2007-06-16

**Authors:** Zhipeng Cai, Randy Goebel, Mohammad R Salavatipour, Guohui Lin

**Affiliations:** 1 Department of Computing Science, University of Alberta. Edmonton, Alberta T6G 2E8, Canada

## Abstract

**Background:**

Gene expression microarray is a powerful technology for genetic profiling diseases and their associated treatments. Such a process involves a key step of biomarker identification, which are expected to be closely related to the disease. A most important task of these identified genes is that they can be used to construct a classifier which can effectively diagnose disease and even recognize the disease subtypes. Binary classification, for example, diseased or healthy, in microarray data analysis has been successful, while multi-class classification, such as cancer subtyping, remains challenging.

**Results:**

We target on the challenging multi-class classification in microarray data analysis, especially on the cancer subtyping using gene expression microarray. We present a novel class discrimination strength vector to represent individual genes and introduce a new measurement to quantify the class discrimination strength difference between two genes. Such a new distance measure is employed in gene clustering, and subsequently the gene cluster information is exploited to select a set of genes which can be used to construct a sample classifier.

We tested our method on four real cancer microarray datasets each contains multiple subtypes of cancer patients. The experimental results show that the constructed classifiers all achieved a higher classification accuracy than the previously best classification results obtained on these four datasets. Additional tests show that the selected genes by our method are less correlated and they all contribute statistically significantly to the more accurate cancer subtyping.

**Conclusion:**

The proposed novel class discrimination strength vector is a better representation than the gene expression vector, in the sense that it can be used to effectively eliminate highly correlated but redundant genes for classifier construction. Such a method can build a classifier to achieve a higher classification accuracy, which is demonstrated via cancer subtyping.

## 1 Background

DNA microarray technology enables the measurement of expression levels of thousands of genes simultaneously. This unique feature has a fundamental role in a wide range of current biological and medical research. One of the most common applications is to compare the gene expression levels in tissues extracted from different conditions, such as healthy versus diseased tissues, and thus to characterize the genetic profiles of these conditions. Two main genetic profiling tasks have been investigated extensively in the past two decades: identification of biomarker genes, which are the genes differentially expressed under various experimental conditions; and the construction of classifiers based on these identified genes, to effectively recognize the experimental conditions. For example, recognizing the primary anatomical site of tumor origin is a fundamental requirement for optimal treatment for cancer patients.

Early research on genetic profiling employed gene and sample class simultaneous clustering to recognize expression patterns associated with samples inside a class, as well as those genes whose expression levels characterize the class [1,2]. Nevertheless, in general, among the thousands of genes examined, only a small number of them are significantly associated with the experimental conditions. These genes, or biomarkers, would have their expression levels increase or decrease under certain conditions, compared to normal expression levels. Such discriminatory genes are very important in genetic disease studies as they often are set as targets for drug design [3-5]. However, identification of discriminatory genes is not an easy task, because there are usually only a small number of experiments (chips, arrays, or samples) available for a typical application, largely due to the high experimental cost. The huge number of genes versus a tiny number of experiments is a familiar machine learning challenge, often labeled as "the curse of dimensionality".

There are many algorithms proposed in the past two decades for gene selection, most of which directly extend ideas in the more general problem of feature selection/extraction, which is a long-standing research topic in statistics and machine learning. Nevertheless, one should keep in mind that gene selection in gene expression microarray data analysis differs from the general feature selection in many aspects, such as the dimensionality issue and the subsequent data overfitting; consequently, many general feature selection algorithms will not work satisfactorily on microarray data. On the other hand, gene selection is closely related to the other main task in genetic profiling: to build a good sample classifier. Besides validating selected genes via other biological experiments, for example in which they are set as drug targets, another way to confirm that the selected genes are biologically correlated to various experimental conditions is to use them to build a sample classifier and to test its classification accuracy. Clearly, high classification accuracy suggests the good quality of the selected genes. In fact, much research has followed this rule to compare their gene selection methods, in addition to considering the detailed biological annotations for the selected genes.

To name a few gene selection methods, Golub *et al*. [3] developed a measure of correlation that emphasizes the "signal-to-noise" (S2N) ratio in using the gene as a class membership (acute myeloid leukemia or acute lymphoblastic leukemia) predictor, and selected a number of top ranked genes as discriminatory genes. The S2N ratio captures the basic rule of gene selection: a discriminatory gene must have close expression levels in samples within a class, but significantly different expression levels in samples across different classes. Other approaches that follow the same rule, with certain modification and refinement, include t-test [4], regularized t-test [6], supervised gene shaving [2], a method focusing on expression homogeneity in a certain class [7], a method which uses both small within-class expression variance and large between-class expression variance [5], as well as many other so-called single gene scoring or univariate methods.

To further exploit the correlations amongst genes, Xiong *et al*. [8] first ranked genes using their individual classification accuracy and then selected a subset of genes with an overall near maximal classification accuracy through sequential (floating) forward selections (SFS, SFFS). Guyon *et al*. [9] did not rank genes but suggested a gene selection method to use support vector machines (SVMs) combined with recursive feature elimination (RFE) to select a subset of genes with an overall near maximal classification accuracy. Li *et al*. [10] combined a genetic algorithm (GA) and a *k*-nearest neighbor (KNN) method to identify a fixed number of genes that can correctly classify binary samples, based on the occurrence frequency of the gene in many gene subsets. Lee *et al*. [11] and Zhou *et al*. [12] proposed to first constrain the number of genes to be selected and then use a Markov Chain Monte Carlo (MCMC) based stochastic search algorithm to discover important genes. Shevade and Keerthi [13] proposed a logistic regression for class membership estimation based on a linear combination of all the genes, then further reduced the density by setting up a sensitivity threshold to prune genes. The resulting regression model is thus sparse and the remaining genes are considered informative. Díaz-Uriarte and Alvarez de Andres [14] defined a measure of gene importance with the random forest and iteratively removed the gene with the smallest importance until the smallest out-of-bag error rate is yielded.

Jaeger *et al*. [15] proposed a pre-filtering approach where fuzzy clustering was applied, using the gene expression levels across all samples, followed by selecting a varying number of representatives per cluster.

These representative genes were then ranked by the aforementioned t-test to select the top-ranked as feature genes. A similar approach was proposed by Hanczar *et al*. [16] to cluster genes using their expression levels across all samples and then to represent the clusters using their mean expression levels (called *prototype *genes); this was followed with single gene scoring methods on the prototype genes to select a pre-specified number of top-ranked genes. Noticing that in general the gene clustering algorithms need the number of clusters as an input parameter (which is often difficult to tune), the HykGene proposed by Wang *et al*. [17] first applied single gene scoring methods to select a set of top-ranked genes, then performed a hierarchical clustering on them, and lastly selected one representative per cluster to form the final marker genes. The number of clusters in HykGene was determined using the leave-one-out cross validation classification accuracy.

It is worth pointing out that most of the proposed gene selection methods are classifier independent, or they can be combined with any classification methods, such as linear discrimination analysis [4,7,8,14], nearest neighbor models [4,5,10,14,17], support vector machines [5,8,9,14-17], and logistic regression models [8]. These gene selection methods typically produce a small set of biomarker genes which can be used in classifier construction. However, there are some other gene selection methods which are bound with specific classifiers [11-14].

It is also equally important to point out that most of these gene selection methods, and the associated classifiers, work only on two-class datasets, though through one-versus-all (OVA) they can be theoretically extended to multi-class datasets. The methods that have been explicitly tested on multi-class dataset(s) include [4,5,7,14,16,17]. In addition, Ramaswamy *et al*. [18], Su *et al*. [19], and Pomeroy *et al*. [20] specifically dealt with classification of multiple classes of human tumors through identification of a set of tumor genes (see Results for more details).

In the context of gene selection and the subsequent classification, the performance of one method is normally validated through a training stage that tunes the parameters in the method, followed by a testing stage which estimates the quality of the selected genes and the performance of the resultant classifier. For some of the above mentioned methods, such as the ones in [3] and [18], a given gene expression dataset is partitioned into two parts, one called *training dataset *and the other *testing dataset*. The class memberships of training samples are used in the training process while the class memberships of testing samples are blinded to the classifier for estimating its classification accuracy. Other methods adopt cross validation schemes for performance evaluation. There are two popular cross validation schemes, one is ℓ-fold and the other is leave-one-out (LOOCV). In ℓ-fold cross validation [4,5,7], the whole given dataset is (randomly) partitioned into ℓ equal parts, and (ℓ - 1) parts of them are used to form the training dataset while the other one to form the testing dataset; the process is done when every part has been used as a testing dataset. The classification accuracy is defined as the ratio between the number of correctly predicted samples and the total number of testing samples. Most of the methods that adopt this cross validation scheme repeat the (random) partition several times and the average classification accuracy is reported. However, most of the methods, for example, Ramaswamy *et al*. [18], Su *et al*. [19], and Wang *et al*. [17], adopt the LOOCV scheme, in which only one sample is used as the testing sample while all the others are used to form the training dataset; the scheme goes over every sample in the given dataset. Again, the ratio between the number of correctly predicted samples and the total number of samples is defined as the classification accuracy. In this work, on each real cancer microarray dataset, we adopt the same cross validation scheme to the methods we are comparing with. We also report the LOOCV results on all the datasets and some 5-fold cross validation results.

While binary classification has been extensively explored, multi-class classification remains challenging in microarray data analysis. In this work, we focus on gene selection for the *multi-class classification *and we demonstrate the strength of the proposed method by applying it on cancer subtype identification. Our main goal is to select genes that, as a whole, have superior class discrimination strength. To this purpose, for each gene we define its *class discrimination strength vector*, and based on these vectors we measure the similarity between two genes. Such a similarity measurement is adopted in the *k*-means algorithm to cluster genes. Subsequently, a single gene scoring method is used to rank all the genes. Our method then walks through this ordered gene list and picks up one gene per cluster until a pre-specified number is reached. These selected genes are then used to construct a classifier whose performance is evaluated and measured by the LOOCV (and 5-fold cross validation) classification accuracy. Through experiments on four real multi-class human cancer microarray datasets, we demonstrate that our method can achieve significantly higher classification accuracies than the best previously published. In this sense, our method can serve as a useful addition to existing methods for highly accurate human tumor classification.

## 2 Methods

Assume in the given multi-class microarray dataset there are *n *samples on *p *genes, and these *n *samples belong to *m *classes. In the following, we define a novel vector representation for genes, which can differentiate their class recognition strength.

Let *g*_*ij *_denote the expression level of the *i*-th gene in the *j*-th sample. That is, in the gene expression matrix, each row represents a gene, *G*_*i *_= ⟨*g*_*i*1_, *g*_*i*2_,...,*g*_*in*_⟩, and each column represents a sample. For the *i*-th gene, its mean expression level in the *k*-th class is denoted as *h*_*ik*_, for *k *= 1, 2,...,*m*. The value |*h*_*ik *_- *h*_*il*_| captures the difference between the mean expression levels of the *i*-th gene in the *k*-th class and in the *l*-th class. Obviously, if this value is small, then the *i*-th gene would not be effective in discriminating samples from these two classes, but it could be effective otherwise. Therefore, we define the *class discrimination strength vector *for the *i*-th gene as

*H*_*i *_= ⟨|*h*_*i*1 _- *h*_*i*2_|, |*h*_*i*1 _- *h*_*i*3_|,...,|*h*_*i*1 _- *h*_*im*_|, |*h*_*i*2 _- *h*_*i*3_|, |*h*_*i*2 _- *h*_*i*4_|,...,|*h*_*i*2 _- *h*_*im*_|, |*h*_*i*3 _- *h*_*i*4_|,...,|*h*_*i,m*-1 _- *h*_*im*_|⟩.

Let *d*_1_(*i*_1_, *i*_2_) and *d*_2_(*i*_1_, *i*_2_) denote the Euclidean distances between the *i*_1_-th and the *i*_2_-th genes based on their *G*-vectors and *H*-vectors, respectively. Note that there are *n *entries in the *G*-vectors and *m*(*m *- 1)/2 entries in the *H*-vectors, respectively. We define

d(i1,i2)=d1n+2d2m(m−1)
 MathType@MTEF@5@5@+=feaafiart1ev1aaatCvAUfKttLearuWrP9MDH5MBPbIqV92AaeXatLxBI9gBaebbnrfifHhDYfgasaacH8akY=wiFfYdH8Gipec8Eeeu0xXdbba9frFj0=OqFfea0dXdd9vqai=hGuQ8kuc9pgc9s8qqaq=dirpe0xb9q8qiLsFr0=vr0=vr0dc8meaabaqaciaacaGaaeqabaqabeGadaaakeaacqWGKbazcqGGOaakcqWGPbqAdaWgaaWcbaGaeGymaedabeaakiabcYcaSiabdMgaPnaaBaaaleaacqaIYaGmaeqaaOGaeiykaKIaeyypa0ZaaSaaaeaacqWGKbazdaWgaaWcbaGaeGymaedabeaaaOqaaiabd6gaUbaacqGHRaWkdaWcaaqaaiabikdaYiabdsgaKnaaBaaaleaacqaIYaGmaeqaaaGcbaGaemyBa0MaeiikaGIaemyBa0MaeyOeI0IaeGymaeJaeiykaKcaaaaa@4537@

to be the distance between the *i*_1_-th and the *i*_2_-th genes.

We can calculate the Euclidean distance by Eq. (1) between every pair of genes, and then call the *k*-means algorithm to cluster genes, where the default value for the number of gene clusters *k *is 160 (such a value is set based on extensive empirical study, see Discussion). Essentially, *k*-means is a centroid-based clustering algorithm that partitions the genes into *k *clusters based on their pairwise distances, to ensure that intra-cluster similarity is high and inter-cluster similarity is low.

At the same time, we run a gene ranking method to sort all the genes into a decreasing order of their class discrimination strength. Such a method essentially assigns a score for each gene, which approximates its class discrimination strength. The gene scoring functions can be the classification accuracy of individual genes [8], or can be designed to capture the basic rule that discriminatory genes are those having close expression levels in samples in a common class but significantly different expression levels in samples from different classes [4-7]. The latter category of gene ranking methods are also called *single gene scoring methods*, which do not consider the correlation between genes when assigning the scores. In our study, we adopt the F-test [4,6] and the GS method [5] as our base gene ranking methods.

Walking through the gene order and using the gene cluster information obtained by the *k*-means algorithm, our method picks up a pre-specified number of genes under the constraint that at most *T *genes per cluster are included. For ease of presentation, we call our gene selection method, which targets at selecting genes having dissimilar class discrimination strength, the *Disc*-based method, and use Disc-F-test and Disc-GS to denote the facts that the base gene ranking method is the F-test and the GS method, respectively. We remark that the Disc-based method is generic, in that it can use any other single gene scoring method, such as the Cho's gene ranking method, to create the Disc-Cho's gene selection method.

To computationally evaluate the quality of the selected genes as a whole, we use the classification accuracy of the classifier built on the selected genes, under the LOOCV scheme. In other words, for each gene selection method (in this paper, they are F-test, GS, Disc-F-test, and Disc-GS), the selected genes are used in the *k *nearest neighbor model or the support vector machine model to construct a classifier (the KNN-classifier and the SVM-classifier, respectively), and then use the classifier to predict the class memberships of the testing samples. The percentage of correctly predicted memberships is defined as the classification accuracy of the classifier. Essentially, the KNN-classifier [4] predicts the class membership of a testing sample by a majority vote using its *k *nearest neighbors in the training dataset; the linear kernel SVM-classifier [21,22] finds decision planes to best separate the set of training samples having different class memberships, then uses these planes to predict the class memberships of the testing samples. For each class membership prediction, we have also calculated the associated confidence score as follows. Su *et al*. [19] adopt the *Dixon metric *to assign confidence scores, where the distances between the testing sample and all the training samples are calculated, then the "class distance" between the testing sample and a class is derived to be the average distance over all the distances between the testing sample and all the training samples in that particular class. These class distances are then sorted in increasing order, say for example, *D*_1 _≤ *D*_2 _≤...≤ *D*_*m *_where *m *is the number of classes, to compute the value *c *= (*D*_2 _- *D*_1_)/(*D*_*m *_- *D*_1_) which is the confidence value for assigning the closest class to the testing sample. A Dixon threshold of 0.1 is generally accepted as conservative boundary for high confidence prediction. Associated with our KNN-classifier for *k *= 5, assuming the closest 5 neighbors for the testing sample are at increasing distances *d*_1_, *d*_2_,...,*d*_5 _and the *k*_1_-, *k*_2_-, *k*_3_-th neighbors form a majority vote, then (1dk1+1dk2+1dk3)/(1d1+1d2+1d3+1d4+1d5)
 MathType@MTEF@5@5@+=feaafiart1ev1aaatCvAUfKttLearuWrP9MDH5MBPbIqV92AaeXatLxBI9gBaebbnrfifHhDYfgasaacH8akY=wiFfYdH8Gipec8Eeeu0xXdbba9frFj0=OqFfea0dXdd9vqai=hGuQ8kuc9pgc9s8qqaq=dirpe0xb9q8qiLsFr0=vr0=vr0dc8meaabaqaciaacaGaaeqabaqabeGadaaakeaacqGGOaakdaWcaaqaaiabigdaXaqaaiabdsgaKnaaBaaaleaacqWGRbWAdaWgaaadbaGaeGymaedabeaaaSqabaaaaOGaey4kaSYaaSaaaeaacqaIXaqmaeaacqWGKbazdaWgaaWcbaGaem4AaS2aaSbaaWqaaiabikdaYaqabaaaleqaaaaakiabgUcaRmaalaaabaGaeGymaedabaGaemizaq2aaSbaaSqaaiabdUgaRnaaBaaameaacqaIZaWmaeqaaaWcbeaaaaGccqGGPaqkcqGGVaWlcqGGOaakdaWcaaqaaiabigdaXaqaaiabdsgaKnaaBaaaleaacqaIXaqmaeqaaaaakiabgUcaRmaalaaabaGaeGymaedabaGaemizaq2aaSbaaSqaaiabikdaYaqabaaaaOGaey4kaSYaaSaaaeaacqaIXaqmaeaacqWGKbazdaWgaaWcbaGaeG4mamdabeaaaaGccqGHRaWkdaWcaaqaaiabigdaXaqaaiabdsgaKnaaBaaaleaacqaI0aanaeqaaaaakiabgUcaRmaalaaabaGaeGymaedabaGaemizaq2aaSbaaSqaaiabiwda1aqabaaaaOGaeiykaKcaaa@56D9@ is assigned as the confidence value. The prediction is considered as highly confident if the value is greater than 0.75. The SVM-classifier [21] we adopt implements a decision directed acyclic graph to combine several binary classifiers into a multiclass classifier and does not produce all the distances for confidence evaluation. We also calculated the covariance of the selected genes, and conducted permutation tests, to measure the extent of dissimilar class discrimination strength of the selected genes as a whole.

## 3 Computational Results

### 3.1 The Carcinomas Dataset

The Carcinomas dataset contains in total 174 samples (U95a GeneChip) in 11 classes: prostate, bladder/ureter, breast, colorectal, gastroesophagus, kidney, liver, ovary, pancreas, lung adenocarcinomas, and lung squamous cell carcinoma, which have 26, 8, 26, 23, 12, 11, 7, 27, 6, 14, 14 samples, respectively [19]. Each sample contains 9,183 genes, whose maximum hybridization intensity is ≥ 200 in at least one sample. All hybridization intensity values < 20 were raised to 20. The data were subsequently log transformed. We obtained this dataset through the website provided by Su *et al*. [19,23].

On this dataset, Su *et al*. [19] applied a Wilcoxon score to rank genes and selected the top ranked 100 genes for each of the 11 classes. Using a subset of 100 samples, under the OVA/LOOCV scheme, an SVM-classifier was used to further select 110 best genes (10 genes per class), among the 1100 genes, which achieved the highest LOOCV classification accuracy (97 out of the 100 predictions were correct; 94 out of the 100 predictions were confident, among which 92 were correct). The classifier trained on these 100 samples was then tested independently on the other 74 samples (no Bladder/Ureter or Pancreas samples), and achieved 70 correct predictions, amongst which 64 were confident.

Our Disc-F-test method was trained on the same 100 samples by setting 160 gene clusters to select 80 genes. The KNN-classifier was able to make 72 correct predictions on the 74 testing samples, among which 71 were confident and among the confident predictions 70 were correct. It was pointed out that the samples not being correctly classified in the original paper are considered very difficult for computational classification [19]. Therefore, our Disc-F-test-KNN classifier can be regarded as a significant improvement (the detailed predictions in Table 1). Alternatively, our Disc-GS-KNN classifier, with exactly the same settings, performed slightly worse than the Disc-F-test-KNN classifier, making only 70 correct predictions out of 74, among which 70 are confident and 68 of them are accurate. Nevertheless, the Disc-GS-KNN classifier also outperformed the method by Su *et al*.

**Table 1:** The detailed predictions on the Carcinomas dataset. Carcinomas dataset: testing result of the Disc-F-test-KNN classifier trained on the 100 samples by setting 160 gene clusters to select 80 genes. 72 out of the 74 predictions are correct (in bold).

# Samples		P	B	C	G	K	LI	O	LA	LS
Prostate (P)	16	**16**								
Breast (B)	14		**13**	1						
Colorectal (C)	12			**12**						
Gastroesophagus (G)	1				**0**		1			
Kidney (K)	1					**1**				
Liver (LI)	1						**1**			
Ovary (O)	18							**18**		
Lung Adeno. (LA)	5								**5**	
Lung Squamous (LS)	6									**6**

**Figure 1:**
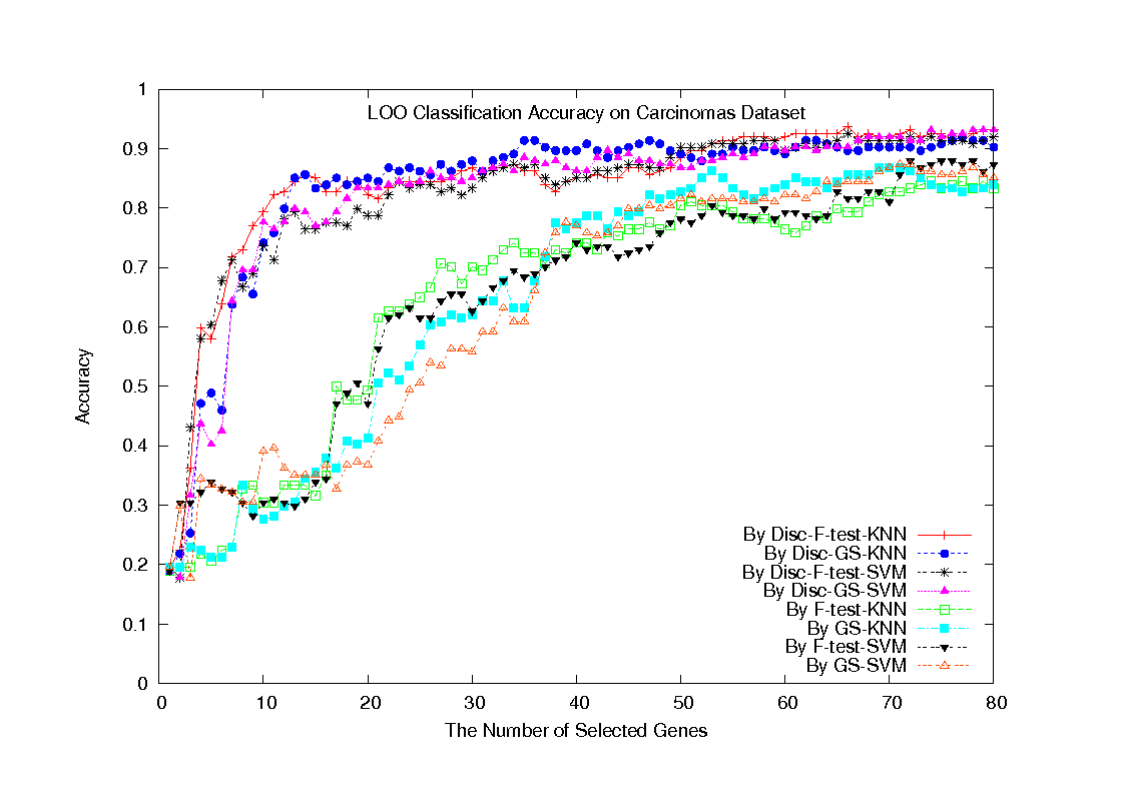
**LOOCV classification accuracies on the Carcinomas dataset**. LOOCV classification accuracies of the eight classifiers on the Carcinomas dataset.

We have also performed a LOOCV on the F-test, GS, Disc-F-test, and Disc-GS methods, combined with the SVM and KNN (*k *= 5) classifiers, on the whole dataset of 174 samples. To run the Disc-based methods, we set the number of gene clusters in the *k*-means algorithm to be 160. Figure 1 plots their classification accuracies with a number of selected genes, ranging from 1 to 80. The plot clearly shows that the Disc-based methods significantly outperformed the single gene scoring methods (*p *= 2.9762 × 10^-8^), in terms of the classification accuracy, but there was a convergence tendency with an increasing number of selected genes. Typically, when 80 genes were selected, the Disc-F-test-KNN classifier made 168 confidence predictions out of 174, among which 159 are correct (confident classification accuracy 94.6%). Among the other 6 non-confident predictions, 4 are correct. The overall classification accuracy is 93.7%.

### 3.2 The Embryonal Dataset

The Embryonal dataset [24] contains a total of 42 patient samples analyzed with oligonucleotide microarrays containing probes for 7,129 genes (HuGeneFL arrays; dataset A in [20]). These 42 samples include 10 medulloblastomas, 5 CNS AT/RTs, 5 renal and extrarenal rhabdoid tumours, and 8 supratentorial PNETs, as well as 10 non-embryonal brain tumours (malignant glioma) and 4 normal human cerebella. Note that there is a slight difference between this dataset and the one mentioned in the original paper [20], which contains 6,817 genes. Note also that CNS AT/RTs and rhabdoid tumours are together considered as a class, so there are 42 samples in 5 separate classes.

On this dataset, Pomeroy *et al*. [20] applied OVA S2N statistics (and the standard t-statistics) to select a number of genes, and then built a weighted KNN (*k *= 5) to predict class memberships. This method obtained 35 correct predictions of the 42 under the LOOCV scheme.

Our Disc-GS-KNN classifier made 36 correct predictions when the number of selected genes was 45 and the number of clusters was set to 100 (Table 2). Note that there is no confidence evaluation associated with the predictions reported in the original paper. For the Disc-GS-KNN classifier, 28 out of the 36 correct predictions were confident. The Disc-F-test-KNN classifier also made 34 correct predictions out of the 42.

**Table 2:** The detailed predictions on the Embryonal dataset. Embryonal dataset: LOOCV result of the Disc-GS-KNN classifier by setting 100 gene clusters to select 45 genes. 36 out of the 42 predictions were correct (in bold).

# Samples		M	CRE	S	MG	N
Medulloblastomas (M)	10	**9**		1		
CNS, renal and extrarenal rhabdoid (CRE)	10	1	**9**			
Supratentorial PNETs (S)	8	2		**6**		
Malignant glioma (MG)	10		1		**9**	
Normal (N)	4			1		**3**

**Figure 2:**
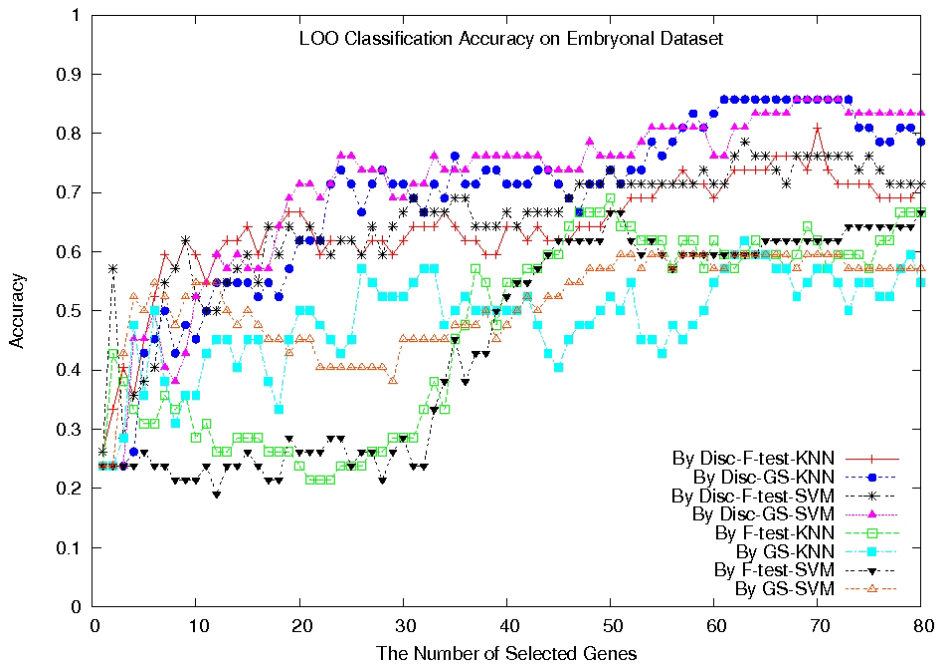
**LOOCV classification accuracies on the Embryonal dataset**. LOOCV classification accuracies of the eight classifiers on the Embryonal dataset.

Again we performed a complete LOOCV on the F-test, GS, Disc-F-test, and Disc-GS methods, combined with the SVM classifier and the KNN classifier, respectively. Since the number of samples in each class is small, we set *k *= 2 in the KNN classifier. To run the Disc-based methods, we set the number of gene clusters in the *k*-means algorithm to be 100, since the number of genes is relatively small compared to the other datasets. Figure 2 plots their classification accuracies when a number of genes are selected, which ranges from 1 to 80. The plot clearly shows that the Disc-based methods significantly outperformed the single gene scoring methods (*p *= 1.1102 × 10^-16^), in terms of the classification accuracy.

### 3.3 The Lung Carcinomas Dataset

The Lung Carcinomas dataset [25] contains 203 samples (U95A oligonucleotide probe arrays; dataset A in [26]) on 12,600 genes. These 203 samples are distributed in 5 classes (Table 3). By removing those genes with standard deviations smaller than 50 expression units, the resultant dataset contains 3,312 genes.

**Table 3:** The detailed predictions on the Lung Carcinomas dataset. Lung Carcinomas dataset: LOOCV result of the Disc-GS-KNN classifier by setting 160 gene clusters to select 80 genes. 197 out of the 203 predictions were correct (in bold).

# Samples		A	SQ	P	SM	N
Adenocarcinomas (A)	139	**137**	1			1
Squamous cell lung carcinomas (SQ)	21	3	**18**			
Pulmonary carcinoids (P)	20			**20**		
Small-cell lung carcinomas (SM)	6				**6**	
Normal (N)	17	1				**16**

Hanczar *et al*. [16] proposed a method that first uses the *k*-means algorithm to cluster genes and then defines a *progene *as the mean expression vector of the genes in a cluster. Subsequently, a single gene scoring method is run on the progenes to select a subset of them to build an SVM-classifier. Under the 3-fold cross validation (averaged over several times of random partition), a highest classification accuracy of 95.2% was obtained in [16], where 100 gene clusters were formed and 100 progenes were selected.

Similarly, under the 3-fold cross validation in which 20 random partitions have been performed, our Disc-GS-KNN and Disc-F-test-KNN classifiers were able to achieve 96.5% classification accuracy, when no more than 80 genes were selected.

**Figure 3:**
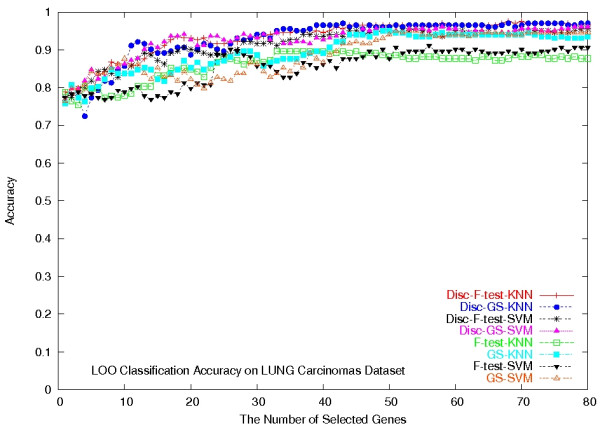
**LOOCV classification accuracies on the Lung Carcinomas dataset**. LOOCV classification accuracies of the eight classifiers on the Lung Carcinomas dataset.

Again we did a LOOCV on the F-test, GS, Disc-F-test, and Disc-GS methods, combined with the SVM classifier and the KNN classifier (*k *= 5), respectively, on the dataset. To run the Disc-based methods, we set the number of gene clusters in the *k*-means algorithm to be 160. Figure 3 plots their classification accuracies with respect to the number of selected genes, ranging from 1 to 80. Their classification accuracies clearly show that the Disc-based methods significantly outperformed the single gene scoring methods (*p *= 1.9447 × 10^-10^), in terms of the classification accuracy. For both Disc-based methods, the KNN classifiers made 200 confident predictions out of the 203, among which 195 were correct (see also Table 3 for the detailed result by Disc-GS-KNN classifier).

### 3.4 The All Tumor Dataset

Ramaswamy *et al*. [18] targeted pure molecular classification of tumor samples and assembled the All Tumor dataset, which contains 218 tumor and 90 normal tissue samples on 16,063 genes (Hu35KsubA oligonucleotide microarrays) [27]. Using these samples, one training dataset consists of 144 tumor samples (8 breast, 8 prostate, 8 lung, 8 colorectal, 16 lymphoma, 8 bladder, 8 melanoma, 8 uterus-adeno, 24 leukemia, 8 renal, 8 pancreas, 8 ovary, 8 mesothelioma, and 16 CNS). The testing dataset consists of the other 46 tumor samples (Table 4).

**Table 4:** The detailed predictions on the All Tumor dataset. All Tumor dataset: testing result of the Disc-GS-KNN classifier by setting 160 gene clusters to select 80 genes. 36 out of the 46 predictions were correct (in bold).

# Samples		BR	PR	LU	C	LY	BL	ML	U	LE	R	PA	O	MS	CNS
Breast (BR)	3	**2**					1								
Prostate (PR)	2		**0**				1								
Lung (LU)	3			**2**							1				
Colorectal (C)	3				**3**										
Lymphoma (LY)	6					**6**									
Bladder (BL)	3						**2**	1							
Melanoma (ML)	2							**2**							
Uterus-adeno (U)	2								**2**						
Leukemia (LE)	6									**5**	1				
Renal (R)	3			1			1				**1**		1		
Pancreas (PA)	3				1							**2**			
Ovary (O)	3						1						**2**		
Mesothelioma (MS)	3													**3**	
CNS	4														**4**

Adopting OVA scheme, the authors proposed to use a linear SVM algorithm (a KNN algorithm was also tested) to recursively eliminate the bottom 10% genes that show low importance. Under the LOOCV scheme, on the training dataset, their method was able to make 115 (or 79.9%) confident predictions of which 103 (or 89.6%) were correct. Only 8 (or 27.6%) of the other 29 (or 20.1%) predictions of low confidence were correct, which gave an overall training classification accuracy of 78.5%. The classifier thus trained on the 144 samples was tested on the independent 46 samples, and achieved a classification accuracy of 78.3% (among which, 30 of the 36 confident predictions were correct, 6 of the 10 low-confidence predictions were correct). The LOOCV classification accuracy of our Disc-GS-KNN classifier on the training dataset reached 78.5% too (the detailed predictions in Table 4), the same as that in [18]. Nonetheless, with respect to confident prediction, our Disc-GS-KNN classifier performed much better on the testing dataset, where it made 41 (or 89.1%) confident predictions, of which 34 (or 82.9%) were correct.

**Figure 4:**
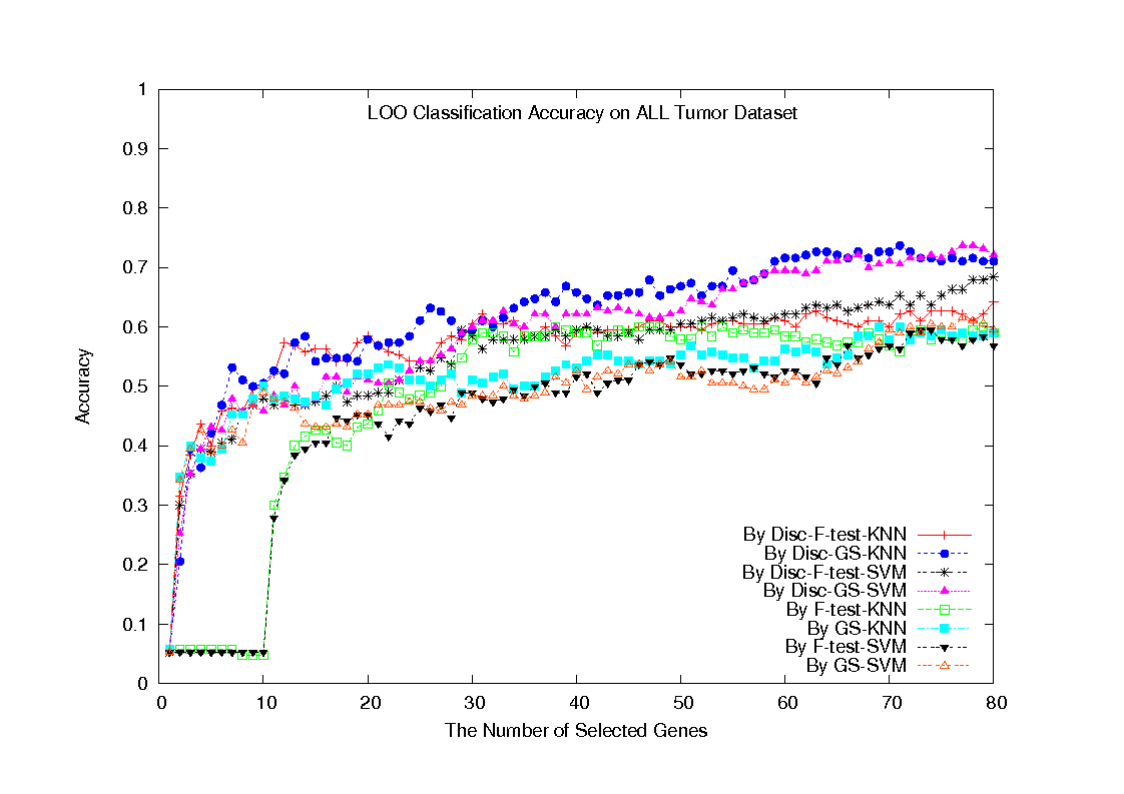
**LOOCV classification accuracies on the All Tumor dataset**. LOOCV classification accuracies of the eight classifiers on the All Tumor dataset.

We have also performed a LOOCV on the F-test, GS, Disc-F-test, and Disc-GS methods, combined with the SVM classifier and the KNN classifier (*k *= 5), respectively. To run the Disc-based methods, we set the number of gene clusters in the k-means algorithm to be 160. Figure 4 plots their classification accuracies with respect to the number of selected genes, ranging from 1 to 80. The plot shows that the Disc-based methods outperformed the single gene scoring methods (*p *= 3.2538 × 10^-8^), typically when combined with the KNN classifier, in terms of the classification accuracy. For both Disc-based methods, when selecting 80 genes, the KNN classifiers made 146 (or 76.8%) confident predictions out of the 190 predictions, among which 124 (or 84.9%) were correct.

## 4 Discussion

### 4.1 The Number of Gene Clusters and the Maximum Number of Genes per Cluster

**Figure 5:**
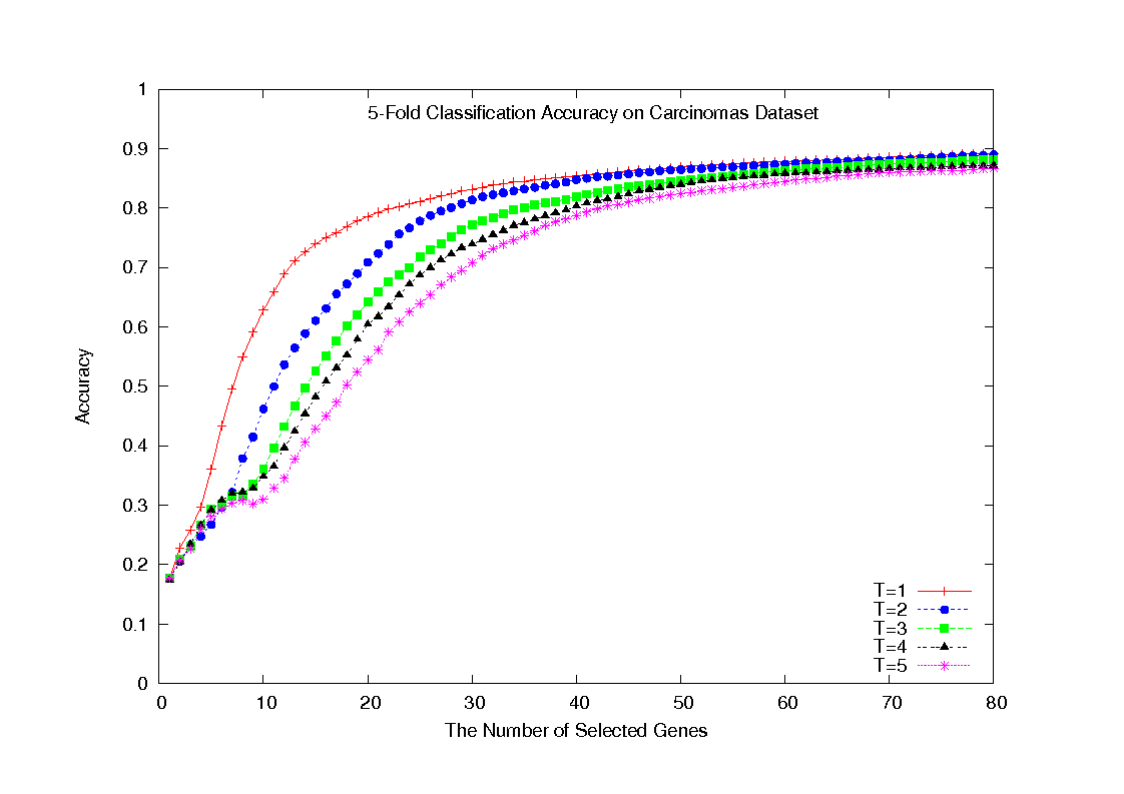
**Average 5-fold cross validation classification accuracies on the Carcinomas dataset**. Average 5-fold cross validation classification accuracy with respect to selecting *T *genes per cluster, on the Carcinomas dataset.

**Figure 6:**
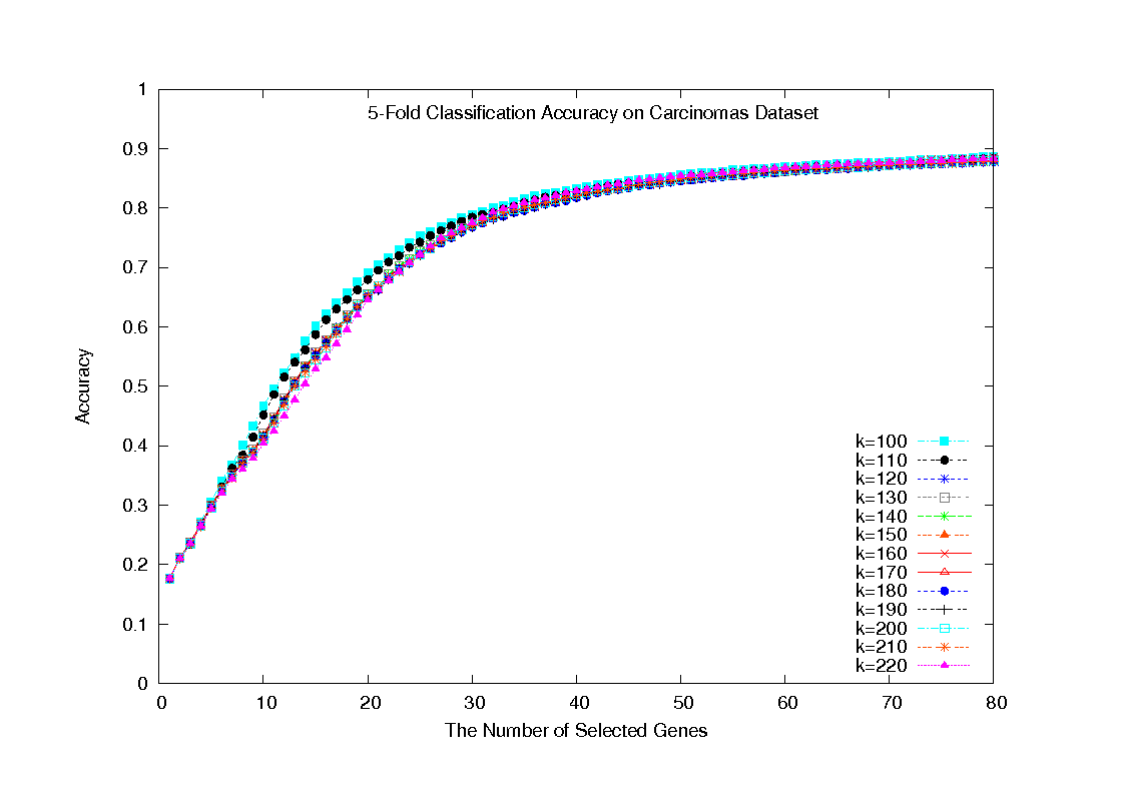
**Average 5-fold cross validation classification accuracies on the Carcinomas dataset**. Average 5-fold cross validation classification accuracy with respect to *k *gene clusters, on the Carcinomas dataset.

Based on the hypothesis that all strong gene clusters must be identified, in order to support reasoning about biologically plausible causation, we have adopted *Principal Component Analysis *and various hierarchical clustering algorithms to find the most likely number of predictive gene clusters in a dataset. Unfortunately, the determination was not uniformly successful, as on some datasets the returned number of gene clusters is unreasonably small (data not shown). Obviously, if too many gene clusters are created, the Disc-based gene selection methods might still include too many redundant genes. On the other hand, if the number of gene clusters is too small, then the Disc-based methods would miss some useful genes. However, the true number of gene clusters must remain dataset dependent, and the effective determination of that number is challenging. We chose to examine several likely values for the number of gene clusters, *k*, in the *k*-means algorithm: *k *= 100 – 220 in tens. For each *k*, we examined at most *T *genes per cluster, for *T *= 1 – 5. For the Carcinomas dataset, all the corresponding classification accuracies, with respect to the number of selected genes, are plotted in Figures 5 and 6. In these plots, we used a 5-fold cross validation scheme (20 random partitions) and combined with the SVM and the KNN classifiers. (That is, each reported classification accuracy was the average over 2000 or 5200 individual classification accuracies, respectively.) Figures 5 and 6 show that the classification accuracy reached the highest when *T *= 1, and the value of *k *had little impact on the experimental results. In fact, the classification accuracies were almost the same at all 13 different values for *k*. We decided on a default setting of *k *= 160 and *T *= 1.

**Figure 7:**
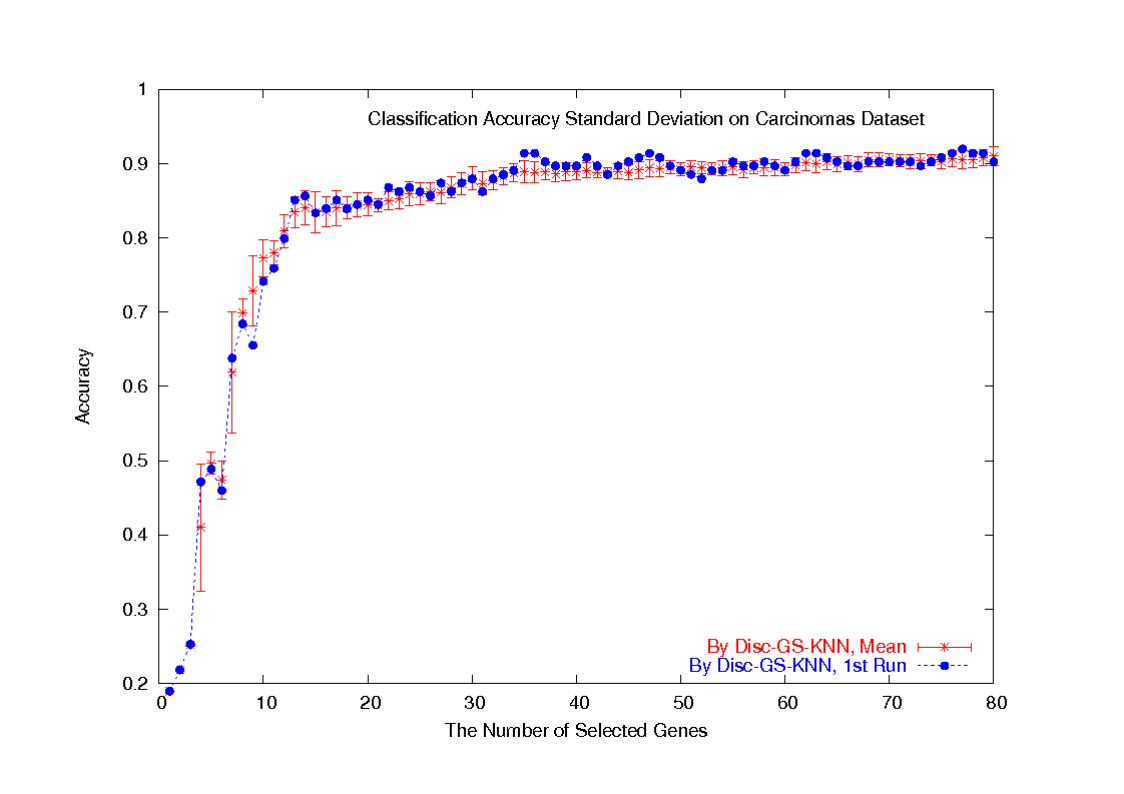
**Standard deviations of the LOOCV classification accuracies on the Carcinomas dataset**. On the Carcinomas dataset, the LOOCV classification accuracies of the Disc-GS-KNN classifier for the first run of the *k*-means gene clustering algorithm plotted together with the average classification accuracies and the standard deviations over 100 runs of the *k*-means algorithm.

Note that we randomly selected *k *genes as initial cluster centers every time we ran the *k*-means algorithm. The reported results in the last section are from the first run of the *k*-means algorithm. We in fact have repeated the whole process 100 times, but we found out that the detailed gene clustering results from the *k*-means algorithm do not affect the subsequent classification accuracies. For the LOOCV study on the whole Carcinomas dataset of 174 samples, the standard deviations of the classification accuracies of the four Disc-based classifiers are all very small across all the numbers of selected genes, and the reported classification accuracies of the four Disc-based classifiers on the first run of the *k*-means algorithm all have *p *values around 0.5, indicating that they are not particularly correlated to the *k*-means gene clustering results. For the Disc-GS-KNN classifier, the LOOCV classification accuracies on the first run of the *k*-means gene clustering algorithm are plotted in Figure 7, together with the average classification accuracies and the standard deviations over 100 runs of the *k*-means algorithm. For the other three Disc-based classifiers, the results are similar and we omit them from Figure 7 since plotting them all makes the figure difficult to read.

### 4.2 The Impact of Class Discrimination Strength Vector

**Figure 8:**
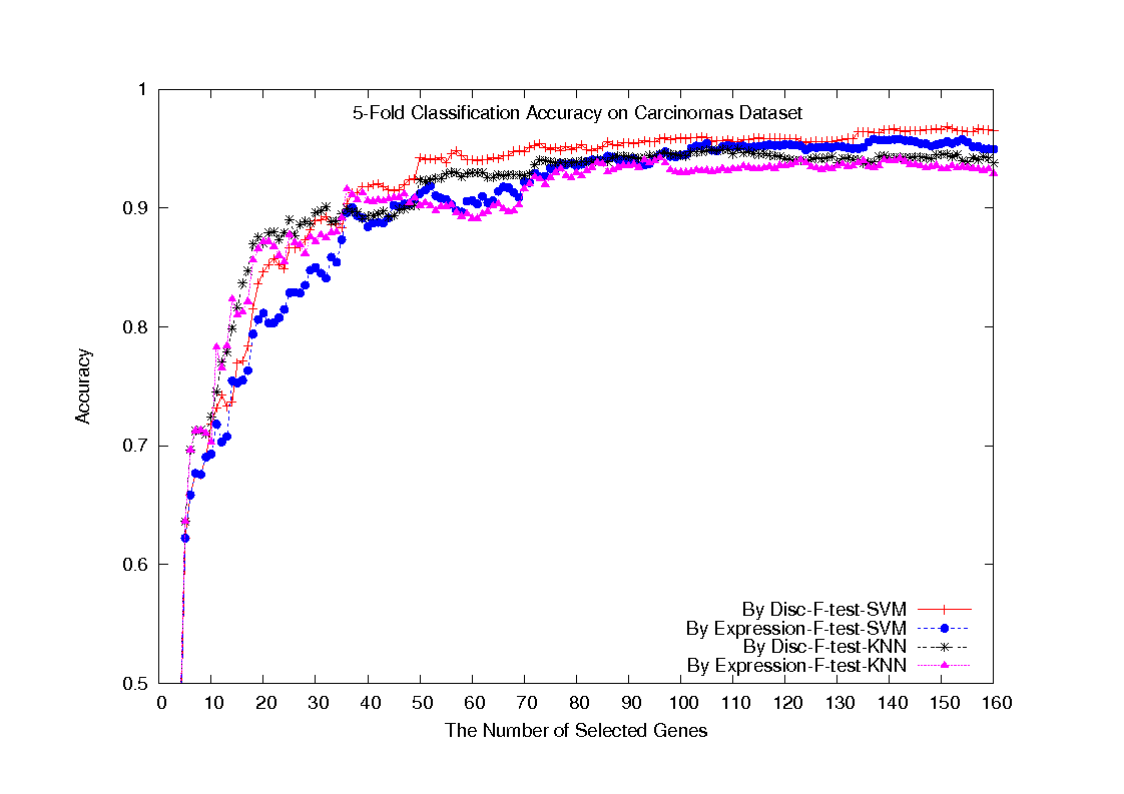
**5-fold cross validation classification accuracies on the Carcinomas dataset**. 5-fold cross validation classification accuracies of the KNN- and SVM-classifiers, where genes are selected using *d*(·, ·) and *d*_1_(·,·) distances combined with the F-test method, on the Carcinomas dataset.

To increase our confidence on the independence of each identified gene cluster and improve accuracy in sample classification, we define the notion of a class discrimination strength vector for each gene, the *H*-vector, which contains the absolute differences between all pairs of class mean expression values. Subsequently, the distance between two genes, *d*(·,·), is defined as a weighted linear combination of the Euclidean distances, *d*_1_(·,·) and *d*_2_(·,·) between their *G*-vectors and their *H*-vectors (see Methods), respectively. For comparison purpose, we have also experimented with the distance *d*_1_(·,·) in the *k*-means algorithm, and the results showed that using the *H*-vectors does improve the clustering quality, which in turn results in a higher classification accuracy. On the Carcinomas dataset, applying the F-test method to rank all the genes, and then using the gene cluster information returned by both distance measures (160 gene clusters were formed), *d*(·,·) and *d*_1_(·,·), to select one gene per cluster, we collected the 5-fold classification accuracies for both the KNN- and SVM-classifiers and plotted them in Figure 8, where "Disc" and "Expression" indicate the gene cluster information by the distance measures *d*(·,·) and *d*_1_(·,·), respectively.

**Figure 9:**
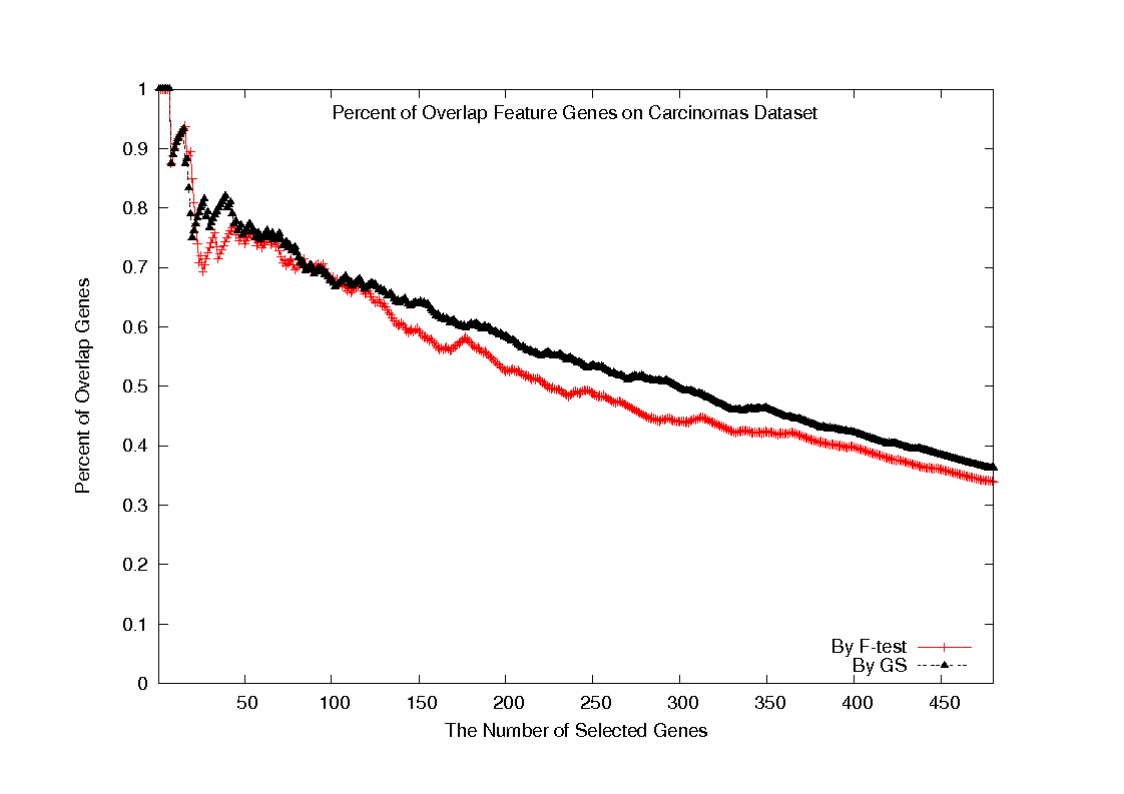
**The percentage of the overlapping genes selected on the Carcinomas dataset**. The percentage of the overlapping genes selected using *d*(·,·) and *d*_1_(·,·) distances combined with the F-test and GS methods, on the Carcinomas dataset.

We have also looked into the percentage of the overlapped genes selected using two different gene cluster information. To this purpose, we allowed minor rank difference to set *T *= 3, that is, up to three genes per cluster were selected. In Figure 9, the *x*-axis labels the total number of selected genes and the *y*-axis labels the percentage of the overlapped genes between the gene sets selected using the distance measures *d*(·,·) and *d*_1_(·,·), respectively, where each of the F-test and the GS methods was applied to rank all the genes. For the first 7 genes, the two distance measures voted the same. Though there are several local peaks, the overall tendency of the overlapping percentage was decreasing, which non-surprisingly indicates that the *H*-vector did contribute to the gene clustering quality that eventually improved the classification accuracy.

### 4.3 Covariance of Selected Genes and Their Permutation Tests

As we mentioned earlier, the underlying design idea in the Disc-based gene selection methods is to avoid selecting too many similar genes for classifier construction. Covariance can be used to measure the correlation among two or more sets of random variables, where a larger covariance value indicates a stronger relationship among the sets of variables. Table 5 shows the average absolute covariance value among all the pairs of gene sets selected by different gene selection methods, on the Lung Carcinomas dataset. In this case, we ran the LOOCV to collect 203 sets of 80 selected genes each. Next, we calculated the frequency of a gene occurring in these 203 sets. The 80 most frequent genes were identified and their covariance was calculated, based on their expression levels across all the 203 samples. The absolute covariance values are listed in Table 5, where one can see that the genes selected by the F-test and GS methods have larger absolute covariance values. This indicates that these two 80-gene sets have stronger relationships to each other, or they share closer expression patterns. The Disc-F-test and Disc-GS methods produced gene sets of smaller covariance values, indicating that the selected genes are more dissimilar to each other.

**Table 5:** Statistical significance analysis of the selected genes on the Lung Carcinomas dataset. Covariance and standard deviation of the genes selected by the Disc-F-test, F-test, Disc-GS, and GS methods, on the Lung Carcinomas dataset.

Method	F-test		GS	
	
	Covariance	Standard Deviation	Covariance	Standard Deviation
Disc-Based	0.3342	0.3851	0.3021	0.3534
Non-Disc-Based	0.7100	0.6502	0.5127	0.5001

For each of these four 80-gene sets, we also examined its quality by doing the permutation test under the 5-fold cross validation scheme. Each time, the sample class labels in the complete dataset were randomly permuted, then the dataset was randomly partitioned into 5 equal parts to build the KNN- and SVM-classifiers using these 80 genes, and lastly the average classification accuracy was collected. The random permutation was repeated for 10,000 times and the 10,000 classification accuracies were fitted into a normal distribution. On the Embryonal dataset, the *p*-values of the achieved classification accuracies on the original dataset, by all eight classifiers, are listed in Table 6, where one can see that the *p*-values associated with the Disc-based methods are much smaller, indicating the higher quality of the selected genes.

**Table 6:** Permutation test result on the Embryonal dataset. The *p*-values of the classification accuracies, under 10,000 permutation tests, of the KNN- and SVM-classifiers built using the 80 genes selected by the Disc-F-test, F-test, Disc-GS, and GS methods, on the Embryonal dataset.

	KNN-Classifier	SVM-Classifier
F-test	6.1533 × 10^-10^	1.9375 × 10^-11^
Disc-F-test	5.1585 × 10^-11^	2.1727 × 10^-13^
GS	1.2128 × 10^-9^	9.0832 × 10^-7^
Disc-GS	1.3323 × 10^-15^	1.0000 × 10^-15^

## 5 Conclusion

In this paper, we aimed at solving the much more challenging multi-class classification problem in microarray data analysis. We have examined a novel method to incorporate gene cluster information to identify many biologically relevant discriminatory genes, and to use that information to construct classifiers for such a purpose. We define a distance measurement between two genes to approximate their difference in the class discrimination strength, based on a novel class discrimination strength vector representation. This Disc-based gene selection method is generic, in that it can be combined with any gene ranking method to identify genes that have dissimilar strength in class discrimination, but together they would have superior class discrimination strength. Our experiments on four real human cancer microarray gene expression datasets showed that the Disc-based gene selection methods achieved significantly higher classification accuracies, compared to the corresponding non-Disc-based gene selection methods.

## 6 Authors' contributions

ZC, MRS, and GL participated in the method design. ZC and GL performed all the experiments. ZC, RG, and GL participated in the experimental result discussion. All authors participated the paper writing. RG and GL finalized the submission. All authors read and approved the final manuscript.
